# Whole Genome Analyses of a Well-Differentiated Liposarcoma Reveals Novel *SYT1* and *DDR2* Rearrangements

**DOI:** 10.1371/journal.pone.0087113

**Published:** 2014-02-05

**Authors:** Jan B. Egan, Michael T. Barrett, Mia D. Champion, Sumit Middha, Elizabeth Lenkiewicz, Lisa Evers, Princy Francis, Jessica Schmidt, Chang-Xin Shi, Scott Van Wier, Sandra Badar, Gregory Ahmann, K. Martin Kortuem, Nicole J. Boczek, Rafael Fonseca, David W. Craig, John D. Carpten, Mitesh J. Borad, A. Keith Stewart

**Affiliations:** 1 Comprehensive Cancer Center, Mayo Clinic, Scottsdale, Arizona, United States of America; 2 Clinical Translational Research Division, Translational Genomics Research Institute, Phoenix, Arizona, United States of America; 3 Department of Biomedical Statistics and Informatics, Mayo Clinic, Scottsdale, Arizona, United States of America; 4 Center for Individualized Medicine, Mayo Clinic, Rochester, Minnesota, United States of America; 5 Department of Health Sciences Research, Mayo Clinic, Rochester, Minnesota, United States of America; 6 Research, Mayo Clinic, Scottsdale, Arizona, United States of America; 7 Hematology, Mayo Clinic, Scottsdale, Arizona, United States of America; 8 Mayo Graduate School, Mayo Clinic, Rochester, Minnesota, United States of America; 9 Division of Hematology/Oncology Mayo Clinic, Scottsdale, Arizona, United States of America; 10 Neurogenomics Division, Translational Genomics Research Institute, Phoenix, Arizona, United States of America; 11 Integrated Cancer Genomics Division, Translational Genomics Research Institute, Phoenix, Arizona, United States of America; Duke-National University of Singapore Graduate Medical School, Singapore

## Abstract

Liposarcoma is the most common soft tissue sarcoma, but little is known about the genomic basis of this disease. Given the low cell content of this tumor type, we utilized flow cytometry to isolate the diploid normal and aneuploid tumor populations from a well-differentiated liposarcoma prior to array comparative genomic hybridization and whole genome sequencing. This work revealed massive highly focal amplifications throughout the aneuploid tumor genome including *MDM2*, a gene that has previously been found to be amplified in well-differentiated liposarcoma. Structural analysis revealed massive rearrangement of chromosome 12 and 11 gene fusions, some of which may be part of double minute chromosomes commonly present in well-differentiated liposarcoma. We identified a hotspot of genomic instability localized to a region of chromosome 12 that includes a highly conserved, putative L1 retrotransposon element, LOC100507498 which resides within a gene cluster (*NAV3*, *SYT1*, *PAWR*) where 6 of the 11 fusion events occurred. Interestingly, a potential gene fusion was also identified in amplified *DDR2*, which is a potential therapeutic target of kinase inhibitors such as dastinib, that are not routinely used in the treatment of patients with liposarcoma. Furthermore, 7 somatic, damaging single nucleotide variants have also been identified, including D125N in the PTPRQ protein. In conclusion, this work is the first to report the entire genome of a well-differentiated liposarcoma with novel chromosomal rearrangements associated with amplification of therapeutically targetable genes such as *MDM2* and *DDR2*.

## Introduction

Adipocytic tumors are classified by the World Health Organization (WHO) International Agency for Research on Cancer (IARC) into benign, intermediate and malignant classes [1]. Intermediate tumors include atypical lipomatous tumors/well-differentiated liposarcoma (WDLS) that constitute locally aggressive mature adipocytes [1]–[3]. Among those diagnosed with liposarcoma, 40–45% will have WDLS [4], [5]. Surgical removal of the tumor is the primary treatment modality for WDLS as generally WDLS do not respond to chemotherapy and therapeutic options are limited for those with metastatic disease [4], [6]. WDLS occur commonly in the retroperitoneum and in the extremities, but can also occur in the mediastinum and paratesticular region [7]. WDLS tumors in the retroperitoneum or mediastinum are more likely to recur than tumors at other sites with this frequent recurrence resulting in death from local effects of the disease [2], [4]. While WDLS does not typically metastasize, it can dedifferentiate and progress to a more aggressive and potentially metastatic tumor [2], [4].

A number of cytogenetic abnormalities have been associated with WDLS. Supernumerary rings and giant marker chromosomes are the most frequent cytogenetic abnormalities associated with WDLS [8]–[10] that often contain amplifications of chromosome 12, specifically in the 12q13-q15 region [3], [11]. Interestingly, benign lipomas also contain chromosomal rearrangements in the 12q14-q15 region [3], [12]. A number of genes have been identified in these amplified regions including those with a potential oncogenic role including: *MDM2*, *CDK4*, *HMGA2*, and *TSPAN31* [9], [10], [13]–[15]. While amplification of *MDM2* and *CDK4* frequently occur together [9], [10], [13], [15], the amplicons for *MDM2* and *CDK4* have been identified as being separate [13]. Patients with amplification of *MDM2* but no amplification of *CDK4* have a more favorable prognostic outlook than patients with amplifications in both genes [16]. *MDM2* amplification has also been found to occur together with amplification of a neighboring gene, *CPM* [17].

Amplifications have also been found in genes flanking *CDK4* (*STAT6, B4GALNT1, OS9*, *CENTG1, TSPAN31, METTL1* and *XRCC6BP1*) and *MDM2* (*FRS2, CCT2, LRRC10*, and *BEST3*) [15]. Additional genes potentially of interest located in the 12q13-q15 region include: amplified genes *HMGIC* and *GLI*, as well as a non-amplified gene, *CHOP* (also known as *DDIT3*) that is part of mixoid liposarcoma translocations [9], [13], [17], [18]. Amplifications have also been identified in regions 12q21-q22 and associated with overexpression of *CCDC131, GLIPR1, BBS10, ZDHHC17, KITLG* and *WDR51B* [15].

While the above studies have led to a greater understanding of the genetics underlying WDLS, they have not significantly advanced the standard of care for WDLS patients. In order to better understand the genetic basis of disease in liposarcoma, and to identify potential therapeutic targets, we sought to complete whole genome sequencing (WGS) in a WDLS patient. One challenge of studying the liposarcoma genome is the low DNA content in the tissue due to the majority of the cell volume consisting of lipid. As a result, DNA yields are low utilizing standard tumor purification and DNA extraction methods. In order to improve tumor purity and to extract DNA from highly purified tumor cells, we utilized flow cytometry to isolate the diploid and aneuploid populations from the tumor sample prior to array comparative genomic hybridization (aCGH) and whole genome sequencing (WGS) of a WDLS patient. This work revealed 7 damaging single nucleotide variants in 7 genes, massive amplification across multiple chromosomes, massive rearrangement on chromosome 12, the presence of a putative retrotransposon and 11 fusions between genes.

## Materials and Methods

### Samples

Samples were acquired after written informed consent was obtained in compliance with, and approval by, the Mayo Clinic Institutional Review Board. Peripheral blood was acquired for sequencing of the constitutional genome. DNA was isolated from peripheral blood with the Puregene kit (Qiagen) following the manufacturer's protocol. The tumor was acquired from an abdominal mass debulking and flash frozen. The tissue was then minced in DAPI (4, 6-diamindine-2phenylindole dihydrochloride) stock solution at 10 μg/mL, passed through a 40 μM Nylon Cell Strainer filter (BD Biosciences) to disaggregate nuclei and prepare a single particle suspension. Minced and disaggregated nuclei were sorted based on DNA content with the BD Influx™ flow cytometer (BD Biosciences) equipped with UV excitation at 358 nm and emission at 460 nm. This resulted in >95% purity of tumor cells in sorted samples (Figure S1). A minimum of 10,000 events (after exclusion of doublets) were collected for the MultiCycle analysis and a total of 953,000 events collected in three fractions for DNA extraction. Samples were analyzed at rates below 1000 cells/second in order to yield a good signal of discrimination between singlets and doublets. In order to identify the position of the nuclei with the normal diploid amount of DNA, reference cells obtained from normal fibroblast of healthy volunteers were included. DAPI binds stoichiometrically to the DNA. The stained material has incorporated an amount of dye proportional to the amount of DNA. DNA content analysis included determination of the mean channel fluorescence and the coefficient of variation (CV) of the diploid and aneuploid G0/G1 and G2/M peaks. DNA content and cell cycle were analyzed using the software program MultiCycle (Phoenix Flow System). The ploidy of the aneuploid population was 2.3N and included a large (14%) G2/M (4.6N) fraction. DNA extraction was performed separately for each of the sorted aneuploid and diploid populations with the QIAGEN QIAamp DNA Micro Kit according to the manufacturer's protocol. Samples were eluted twice from each column with 100ul of water for a final volume of 200ul. In order to capture residual nuclei and maximize the final volume of genomic DNA, the original microcentrifuge tubes were rinsed with water, pooled and extracted using the protocol above. The products of this second “rescue” extraction were then added to the initial pooled, extracted samples. Following ethanol precipitation the samples were resuspended in water.

### Array Comparative Genomic Hybridization

Array CGH (aCGH) was conducted as described previously [19]. Briefly, prior to hybridization 100 ng of genomic DNA from each sorted fraction and a commercial 46, XX reference (Promega) were amplified with GenomiPhi amplification kit (GE Healthcare). Tumor and control DNA samples were digested with DNAseI and fragmented DNA was labeled using the BioPrime Array CGH Genomic Labeling kit (Invitrogen). Labeled DNA from each sorted fraction was pooled with the labeled DNA from the reference then hybridized to the Agilent 400K Human Genome CGH Microarray according to the manufacturer's protocol.

### Paired End Library Preparation

Paired-end libraries were prepared using NEBNext DNA sample preparation kit following the manufacturer's protocol (New England Biolab). Briefly, DNA was fragmented using the Covaris E210 sonicator to generate double-stranded DNA fragments with a fragment size of 400–500 bp. The ends were repaired, phosphorylated, followed by 3′ end adenylation. Paired end DNA adaptors were ligated and the resulting constructs size selected for ∼500 bp fragments. The excised gel band was purified following manufacturer's protocol utilizing Qiagen Gel Extraction Kits. These fragments were enriched with 12 cycles of PCR. The concentration and size distribution of the libraries was determined on an Agilent Bioanalyzer DNA 1000 chip.

Libraries were loaded onto paired end flow cells and sequenced as 101 by 2 paired end indexed reads on Illumina HiSeq 2000 and base-calling performed using Illumina's RTA version 1.7.45.0.

### Whole Genome Sequencing Analysis

Alignment and variant detection of the WGS reads were performed utilizing TREAT (Targeted RE-sequencing Annotation Tool) [20]. TREAT is an analytical tool that utilizes open source tools in a pipeline that aligns, identifies and annotates variants. Raw sequence reads were aligned to hg18 with Burrows-Wheeler Aligner (BWA). Post-alignment processing included local realignment with Genome Analysis Toolkit (GATK) [21].

Single nucleotide variants (SNV) and insertions/deletions (indel) were detected utilizing GATK [21] and SNVMix [22]. Identified variants were then placed in the custom annotation pipeline and SNV and indel reports created. SNVMix filtered (probability ≥0.8) variant calls from TREAT were used to extract tumor only variants. Annotation of these files utilized SeattleSeq (http://gvs.gs.washington.edu/SeattleSeqAnnotation/) for variant classification, as well as Sorting Intolerant from Tolerant (SIFT) [23] and Polyphen-2 [24] (http://genetics.bwh.harvard.edu/pph2/) for functional impact prediction of the variants. Variants were then visually validated in the Integrative Genomics Viewer (IGV) [25] and any reads with the variant allele present in the normal were removed. Candidate SNV were then selected for validation by capillary sequencing if they were predicted to result in a damaging mutation by SIFT/Polyphen2.

### Detection of Structural Variants

Potential gene fusions were detected with two methods: an in-house computational tool and visual confirmation of CGH breakpoints in the WGS data. Breakpoints for the amplifications observed in the aCGH data were visually confirmed with IGV in the WGS data to determine potential breakpoints and gene fusions. In addition, bioinformatics identified anomalous reads using a sliding window type approach quantifying the number of anomalous reads pointing to a distinct window elsewhere in the genome. Window sizes were based on the insert size. Regions where the reference or germline genome aligns with either a high number of anomalous reads or a high number of poorly mapped reads were ignored. All anomalous read pairs mapping to coding regions were identified as potential fusion genes and were visually confirmed in IGV. Due to the likely presence of double minute chromosomes in this patient, only potential fusion genes that presented with at least two different potential fusion partners were considered for validation.

### Validation Of Structural Variants

Potential fusion genes were then subjected to PCR followed by capillary sequencing to validate the presence of the fusion (Table S1). Fluorescent *in-situ* hybridization (FISH) was performed as previously described [26] to validate amplification of key genes of interest including *MDM2*, *SYT1* and *DDR2*. Bacterial artificial chromosomes mapping to the appropriate region for each gene were identified in the UCSC Genome browser [27] (http://genome.ucsc.edu/.) (Table S2).

### Identification and characterization of LOC100507498

A genome-wide search across species for sequence elements related to LOC100507498 was done using a megablast search against the nonredundant nucleotide database [28]. A selection of unique hits that were highly similar to the LOC100507498 sequence (>90% identity) were translated in all 6 reading frames using transeq [29]. Translated sequences were used to query the Repbase database of repeat element sequences using the repeatmasker algorithm [30]. Nucleotide sequences were also used to identify closely related transposon HMM profiles using dfam_scan.pl [31]. Sequence alignments of LOC100507498 with known L1 elements [32], [33] was done with clustalw to characterize regions of high conservation [34].

### RNA Sequencing

Frozen tumor tissue was cryofractured with the Cryoprep Impactor (Covaris), and lysed in RLT buffer containing 1% beta-mercaptoethanol. Lysate was passed through a Qiashredder column for homogenization followed by the addition of Qiazol lysis buffer to homogenate. Chloroform was added to the homogenate and mixed in Phaselock tubes (5 Prime, Gaithersburg, MD). The tubes were centrifuged at 16,000 g. The aqueous layer was transferred to a new tube, and 70% ethanol added. The sample was transferred to RNeasy spin columns. The columns were washed, and RNA eluted with nuclease-free water.

RNA-Sequencing data was analyzed utilizing the MAP-RSeq pipeline, developed at the Mayo Clinic. Detailed quality control data is generated with RSeQC software [35]. Paired-end reads were aligned by TopHat 2.0.6 [36] against the hg19 genome build using the bowtie1 aligner option [37]. Gene counts were generated using HTseq software (http://www-huber.embl.de/users/anders/HTSeq/doc/overview.html) and gene annotation files were obtained from Illumina (http://cufflinks.cbcb.umd.edu/igenomes.html). Fusions were predicted with the TopHat-Fusion algorithm [38] and analyzed using custom scripts.

### Pathway analysis

Pathway analysis of genes containing amplifications by aCGH, and SNV or fusion genes by WGS was conducted utilizing IPA Core Analysis build 172788 (Ingenuity® Systems, www.ingenuity.com) and MetaCore Pathway Analysis (GeneGo v6.11.41105, www.genego.com). IPA Core analysis was completed utilizing default parameters. MetaCore Pathway Analysis was completed with threshold  = 0 and P-value  = 0.1. P-values in MetaCore were determined utilizing hypergeometric intersection.

## Results

We depict the case of a 72 year-old Caucasian male with a 23-year history of WDLS of the abdomen. The clinical course was characterized by multiple surgical debulking procedures over this period requiring left adrenal gland resection, partial pancreatectomy, large and small bowel resections, abdominal/pelvic lymphadenectomy, left nephrectomy and splenectomy. Post-operative adjuvant radiotherapy was received after two of the eleven surgical resections during this period. No systemic therapy was administered for his extensive disease. Pathological evaluation of the debulked tumor that was used for WGS demonstrated a well-differentiated liposarcoma consistent with prior evaluations. There was evidence of low mitotic activity (<1 mitosis/10 high power field), high adipose content, FNCLCC grade of 1, >90% tumor cellularity but with very low tumor cell/fat nuclear ratio (<1%). At the time of the debulking associated with WGS analysis, it was felt that surgical options would become extremely limited over time due to the decreasing ability to obtain significant debulking and increasing post surgical recovery times. Along with aCGH and WGS performed on the flow sorted nuclei, a commercially available molecular profiling assay (TargetNow®, Caris Life Sciences) was obtained. The TargetNow® assay demonstrated increased levels of topoisomerase I by immunohistochemistry (2+ in 95% of cells analyzed) and no detection of thymidylate synthetase by immunohistochemistry (in 100% of cells analyzed). The patient was treated with leucovorin calcium, 5-fluorouracil, and irinotecan hydrocloride (FOLFIRI) systemic therapy but obtained no clinical benefit and had disease progression. Unfortunately, the patient's clinical condition deteriorated over the ensuing six-month period with protracted debilitation related to post-surgical recovery and comorbidity from unresected tumor that led to his eventual demise.

Whole genome sequencing was conducted on germline and tumor DNA (SRA accession numbers SAMN02214846, and SAMN02214847). An average of 1.7×10^9^ reads/germline sample and 1.4×10^9^ reads/tumor sample were obtained with 1.5×10^9^ (90%) and 1.3×10^9^ (94%) of these reads respectively aligning to the hg18 reference (Table 1). After filtering for reads with a quality score ≥20, coverage was 43X for the germline and 38X for the tumor. Over 2000 somatic single nucleotide variants (SNV) were identified (Table S3) of which 12 were predicted as potentially deleterious by SIFT/Polyphen-2 and seven were validated by capillary sequencing (Table 2). Eight somatic small insertions and deletions (indels) were identified, but none validated by capillary sequencing.

**Table 1:** Metrics and summary statistics.

	Normal	Tumor
Total reads	1676273096	1363626456
Aligned reads	1508120220	1276332896
Percent aligned	90.0%	93.6%
Coverage depth (raw)	50	43
Coverage depth (quality ≥20)	43	38
Total SNV	3865355	3830574
Somatic SNV	NA	2334
Somatic small insertions/deletions	NA	8

**Table 2:** Validated functionally damaging SNV.

Chr	Position (NCBI36)	Allele change	Amino acid change	Gene
2	210491597	G > A	G1704R	UNC80
6	33025040	T > G	K256T	HLA-DMA
10	5129651	A > T	E93V	AKR1C3
11	57183888	C > G	P122A	CLP1
12	79591146	G > A	D125N	PTPRQ
16	69268358	C > T	D253N	MTSS1L
20	49059955	C > T	V110I	KCNG1

**Figure 1:**
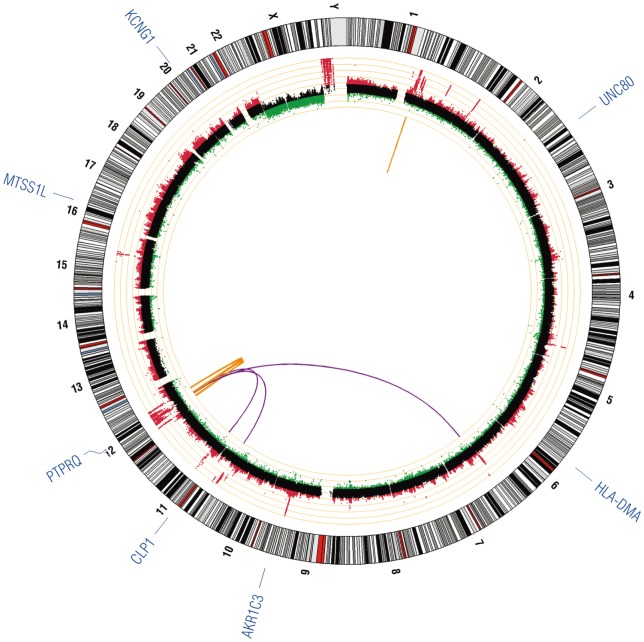
Circos plot of validated genetic variation in a well-differentiated liposarcoma. Inner-most circle contains validated structural rearrangements of fusion genes with translocations indicated in purple, and intra-chromosomal rearrangements indicated in orange. The middle ring contains the aCGH plot with copy number loss indicated in green and copy number gain in red; each orange ring corresponds to a log2 value of 1. The outer-most ring indicates validated, damaging single nucleotide variants.

**Figure 2:**
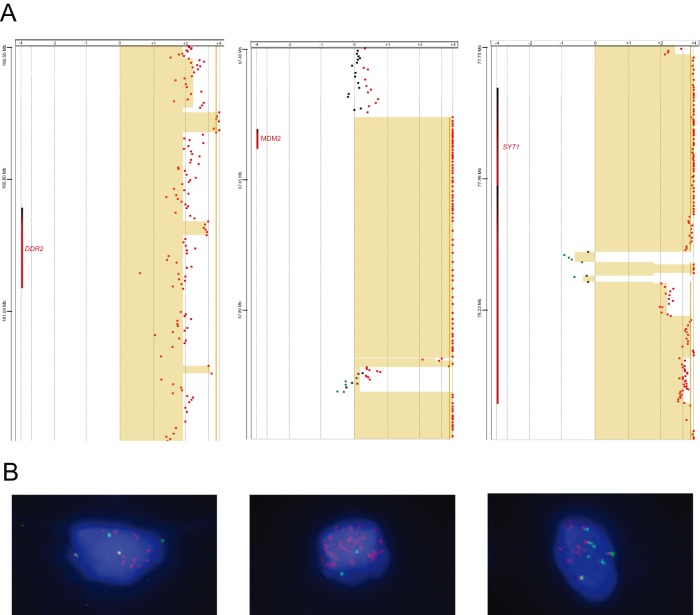
aCGH and Fluorescent *in-situ* hybridization of *DDR2*, *MDM2*, and *SYT1*. (A) aCGH dot plots of the chromosomal region of each gene. Tan colored shading indicates regions of significant copy number change. Red dots indicate copy number gain and green dots indicate copy number loss. (B) FISH images of cells probed for the indicated genes with SpectrumOrange probe and the chromosome control indicated with the SpectrumGreen probe.

Array CGH (GEO accession number GSE47701) revealed massive amplification involving numerous chromosomes. Chromosome 12, which underwent massive rearrangement, was the most significantly affected (Figure 1, middle ring). Breakpoint analysis of these amplified regions in the WGS data revealed 40 potential fusions (Table S4) between genes of which 22 were selected for validation with PCR. Gene fusions validated in DNA by PCR are presented in Table 3. All the genes identified as potential fusion partners were checked in the COSMIC database [39]. None of the genes had reported fusions and all had reported substitution mutations with the exception of *UHMK1*. Amplification of *MDM2* was identified by array comparative genomic hybridization (aCGH) (Figure 2A) and confirmed by FISH (Figure 2B) although we were unable to confirm a potential fusion partner with *MDM2* suggesting the possible presence of *MDM2* on double minute chromosomes. Overexpression of *MDM2* is associated with reduced expression of the key tumor suppressor *TP53* [40]. Interestingly, WGS revealed *SYT1* presenting with three structural rearrangements that include potential fusions with 3 different genes (*FGD6, C12orf26* and *NELL1*) of which 2 are likely part of a ring chromosome due to multiple rearrangements between these genes. RNA Sequencing revealed 17 putative fusion transcripts including a *SYT1*-*C12orf63* fusion (Table S5).

**Table 3:** DNA validated fusion genes.

Chr	Breakpoint 1	Gene 1	Chr	Breakpoint 2	Gene 2	Reads
**Intrachromosomal rearrangements**						
1	160738159	UHMK1	1	160878664	DDR2	Facing
12	70553166	TBC1D15	12	81370954	C12orf26	Same direction – reverse
12	77820664	SYT1	12	94015001	FGD6	Same direction – forward
12	78116536	SYT1	12	81379417	C12orf26	Opposing
12	78600788	PAWR	12	94875477	AMDHD1	Facing
12	81369919	C12orf26	12	94014762	FGD6	Opposing
12	81371116	C12orf26	12	94875230	AMDHD1	Opposing
**Translocations**						
6	157853571	ZDHHC14	12	78591928	PAWR	Same direction – forward
11	21368102	NELL1	12	77114108	NAV3	Same direction – reverse
11	21368605	NELL1	12	78114565	SYT1	Opposing
11	71472153	LRTOMT	12	81346827	C12orf26	Opposing

Furthermore, WGS revealed a gene fusion between *UHMK1* and *DDR2* that was confirmed in DNA, but we were unable to confirm in RNA. Both of these genes are located in a region of copy number gain in this tumor. Copy number gains in 1q23.3 where *UHMK1* and *DDR2* are located, have been reported previously in WDLS [15]. Mutations and increased expression of *DDR2* have been reported in Hodgkin's Lymphoma and anaplastic large cell lymphoma [41], lung squamous cell carcinoma [42], nasopharyngeal carcinoma [43], sarcoma [44], hepatocellular carcinoma [45], aneuploid papillary thyroid cancer [46] and non-small cell lung cancer [47]. *DDR2* is of interest both mechanistically and therapeutically. It plays key roles in multiple cellular activities, including proliferation, migration, adhesion, and extracellular matrix remodeling [48], [49]. The kinase domain of DDR2 is predicted to remain intact and the presence of copy number gain is significant because DDR2 kinase activity has been inhibited with the kinase inhibitors imatinib, nilotinib and dasatinib [50]. Interestingly, the gene fusion event between *UHMK1* and *DDR2*, located in a region of copy number gain, may disrupt the normal function of the *UAP1* gene, encoding a UDP-N-acetylglucosamine pyrophosphorylase. The *UAP1* gene is located between *UHMK1* and *DDR2*, thus structural rearrangement between *UHMK1* and *DDR2* is likely disruptive to the normal function of *UAP1*. The UAP1 protein is predicted to interact with glucosamine (UDP-N-acetyl)-2-epimerase/N-acetylmannosamine kinase (GNE) [51], [52], a kinase regulating the biosynthesis of sialic acids which play a critical role in cell adhesion, signal transduction and tumor metastasis [53]. We find that GNE also resides in an amplified region.

**Figure 3:**
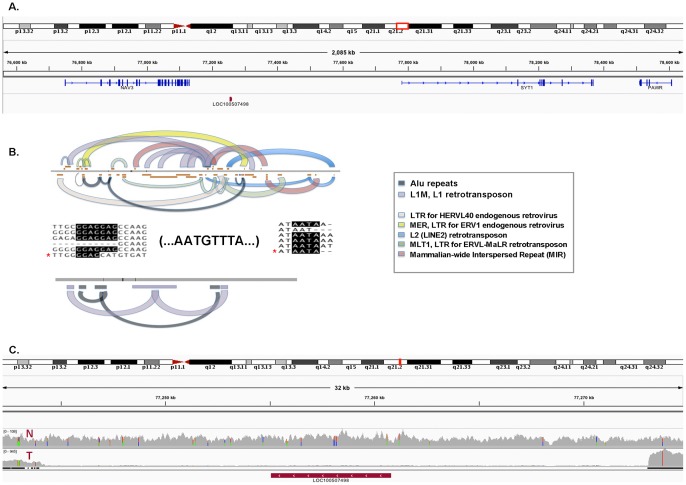
Depiction of genomic rearrangement hotspot on chromosome 12. We identified and further characterized a putative transposable element (LOC100507498) located on the (-) strand, within the PAWR-SYT1-NAV3 gene cluster (**3A**). The LOC100507498 and closely related sequences were characterized by comparing both nucleotide (**3B**,top) and translated (**3B**,bottom) sequences to known families of repetitive elements (Methods). Highly conserved sequence domains/motifs are color coded by known families of repetitive elements (Legend). Overall, these sequences exhibited the highest similarity to the L1 retrotransposon and Alu repeat elements (domain hit counts and similarity score). Sequence alignments of LOC100507498 (*) with known L1 elements [32], [33] exhibited the highest overall homology to Class 3 L1 elements as described by Pickeral et al. (Table 1, [32]) and in addition to the 5′-GGAG and 3′-AATA signature motifs, LOC100507498 carries several ‘AATGTTTA’ motifs that suggest multiple rounds of L1-mediated transduction [33]. The LOC100507498 locus resides within a genomic region that is deleted in the Tumor (T) sample, but present in the Normal (N) genome (**3C**).

In depth analysis of a region of genomic instability hotspot on chromosome 12 revealed the presence of a highly conserved, putative L1 retrotransposon element, LOC100507498 (Figure 3A). This noncoding ∼33 kb RNA nucleotide sequence resides on the minus strand within the *NAV3-SYT1-PAWR* gene cluster on chromosome 12, in which 6 of the 11 validated gene fusion events occur. Two pseudogenes with homology to a mitochondrial ribosomal protein L11 (*MRPL11P3*) and a *RAS* related leukemia viral oncogene (*LOC642550*) also reside within the *NAV3-SYT1-PAWR* gene cluster, proximal to the *LOC100507498* transposon. Characterization of *LOC100507498* and closely related nucleotide (Figure 3B, top) and translated sequences (Figure 3B, bottom) show the highest similarity to L1 retrotransposon and Alu elements. L1 retrotransposons are non-LTR (non-Long Terminal Repeat) elements that have greatly expanded the human genome by autonomous retrotransposition, as well as non-autonomous retrotransposition of other mobile elements (e.g. Alu) which do not have their own transposases [54]. Sub-sequences of the LOC100507498 element were highly conserved (>95% similarity) in other species including Pan Troglodytes, Pan Paniscus, Gorilla, Macaca mulatta, and Nomascus leucogenys. Sequence alignment comparisons of the LOC100507498 element with known L1 retrotransposons showed highest overall conservation with Class 3 L1's (Table 1, [32]) known to be associated with 3′ transduction. A genomic deletion present specifically in patient tumor samples was identified by sequence read alignments to the LOC100507498 locus and surrounding region, indicating that this locus is a hotspot of genomic instability (Figure 3C).

SNV analysis by SIFT/Polyphen-2 revealed 7 SNV in 7 genes with potentially damaging functional significance (Figure 1, Table 2): *AKR1C3, CLP1, HLA-DMA, KCNG1, MTSS1L, PTPRQ*, and *UNC80*. Interestingly, *AKR1C3* is upregulated in castration resistant prostate cancer [55]. All but one of these genes (*HLA-DMA*) have reported somatic mutations in the COSMIC database [39], but none of our SNV are shared and none of these genes have reported mutations in sarcomas.

In order to determine molecular pathways shared by genes containing mutations or copy number gains, pathway analysis was conducted. This analysis revealed expected pathways involved in cell cycle regulation, proliferation, survival, and cellular assembly as well as DNA replication, recombination and repair (Tables 4 and 5). Interestingly, both IPA and MetaCore identified lipid metabolism in their top eight pathways.

**Table 4:** IPA pathway analysis of genes containing SNV, gene fusions or amplifications.

ID	Pathways	Score	Number of focus molecules	Genes from dataset in network
1	Cell Cycle, Cellular Assembly and Organization, DNA Replication, Recombination, and Repair	49	27	AGAP2, AGPS, AIM2, ANAPC10, CCT2, CDK4, CUL1, DYRK2, E2F7, EGLN1, GRIP1, HELB, HMGA2, IFI16, KIF5A, MARS, MDM2, OS9, PNKD, PRRC2C, PYHIN1, RFTN1, SDHC, UHMK1, USP21, USP44, USP9Y
2	Cell-To-Cell Signaling and Interaction, Molecular Transport, Small Molecule Biochemistry	41	23	AKR1C3, ARHGAP9, CRP, DUSP6, F11R, FCER1A, FCER1G, FCGR2A, FCGR2B, FCGR3B, FCRLA, FCRLB, KLF13, NTN4, PAWR, PPFIA2, PTPRB, PTPRD, PTPRH, PTPRR, RGS4, RGS5, STK24
3	Cell-To-Cell Signaling and Interaction, Nervous System Development and Function, Post-Translational Modification	38	22	AP2A2, caspase, CLP1, CPSF6, CYP27B1, FRS2, GAS2, MLL2, NCOR2, NLGN1, NR1I3, NR2C1, NRXN1, NUMA1, PFDN2, SYT1, TAB2, TBL1Y, UBAC1, VAMP4, ZDHHC17, ZFPM1
4	Cell Morphology, Cellular Assembly and Organization, Connective Tissue Disorders	27	17	APOA2, AVIL, CSRP2, DDIT3, DEDD, ESPN, GDA, HLA-DMA, LAMA5, LYZ, MYOC, NELL1, OSBPL8, RGMA, RRBP1, SACS, SGSM2,
5	Connective Tissue Disorders, Developmental Disorder, Skeletal and Muscular Disorders	25	16	ADAMTS4, AKAP6, BAIAP2, DCTN2, DDR2, DISC1, GLI3, HERC2, KCNC2, KDM5D, MPZ, MRC2, NIT1, RAB3IP, SHC2, ZIC3
6	Cellular Compromise, Cellular Function and Maintenance, Small Molecule Biochemistry	23	15	CADM3, CAMSAP1, DDX3Y, DERL3, FGD6, KCNG1, METTL1, NACC2, NAV3, PPOX, RSF1, SCAPER, TOX2, TSFM, UFC1
7	Lipid Metabolism, Molecular Transport, Ophthalmic Disease	21	14	ATP11A, BBS10, CHD2, DNM3, EHD2, FANCF, LCN1, NUF2, PIP4K2C, PTK7, SASH1, STXBP5, TBC1D15, TOMM40L
8	Cell-To-Cell Signaling and Interaction, Infectious Disease, Inflammatory Response	21	14	C11orf67, CCDC41, CCDC94, CPM, GALNTL4, INTS4, KCNT1, OR10J1, PRKRIR, SBNO2, SEPT9, USF1, XRCC6BP1, ZDHHC14

**Table 5:** GeneGo pathway analysis of genes containing SNV, gene fusions or amplifications.

Network #	Pathways	Total	In Data	pValue	Genes from Active Data
1	Development Hedgehog signaling	46	4	2.991E-04	Cullin 1, GLI-3R, DYRK2, GLI-3
2	Cell cycle Influence of Ras and Rho proteins on G1/S Transition	53	3	6.183E-03	CDK4, MLCP (reg), MDM2
3	Cytoskeleton remodeling Neurofilaments	25	2	1.337E-02	Kinesin heavy chain, DCTN2
4	Cell cycle Role of SCF complex in cell cycle regulation	29	2	1.777E-02	Cullin 1, CDK4
5	Regulation of lipid metabolism RXR-dependent regulation of lipid metabolism via PPAR, RAR and VDR	30	2	1.896E-02	CAR, NCOA2 (GRIP1/TIF2)
6	DNA damage ATM/ATR regulation of G1/S checkpoint	32	2	2.142E-02	CDK4, MDM2
7	LRRK2 in neurons in Parkinson's disease	33	2	2.270E-02	AP-2 alpha subunits, AP2A2
8	Cell cycle Spindle assembly and chromosome separation	33	2	2.270E-02	NUMA1, DCTN2
9	Cell cycle ESR1 regulation of G1/S transition	33	2	2.270E-02	Cullin 1, CDK4
10	Signal transduction Erk Interactions: Inhibition of Erk	34	2	2.401E-02	MKP-3, PTPRR

## Discussion

Previous studies in liposarcoma have contributed significantly to the understanding of the genetics underlying WDLS, but none have evaluated these in the context of the entire genome. This work reports the use of flow cytometry to isolate tumor cells from a WDLS prior to whole genome sequencing. Structural rearrangements potentially contributing to tumor development were detected in addition to identification of potential therapeutic targets of interest.

The presence of LOC100507498 with high similarity to L1 retrotransposon and Alu elements in the *NAV3-SYT1-PAWR* gene cluster that was prone to massive rearrangement has potentially significant functional consequences. First, although the majority of L1 and Alu elements are inactive sequence relics of ancient evolutionary events [54], many are still active during development and cancer [54], [56]. Second, in addition to mediating genomic rearrangements, the presence of L1 retrotransposons, which preferentially act in cis [57], can impact genomic stability and gene expression of neighboring genes via a number of different mechanisms [56]. The E2F7 transcription factor that plays an important role in cell cycle regulation [58], [59], is 5′ of the gene cluster, and is in cis with the L1 retrotransposon on the minus strand. Furthermore, the gene protein tyrosine phosphatase receptor type Q (*PTPRQ*) that has been shown to regulate adipogenesis in mesenchymal stem cells [60], resides just 3′ of the *NAV3-LOC100507498-SYT1-PAWR* gene cluster. Interestingly, a related protein tyrosine phosphatase, *PTPRM*, has been identified as an insertional mutagenesis target by L1 retrotransposons in colon tumors [56].

The role of transposons in cancer screening [61], [62] as well as gene therapy [63], [64] has expanded over recent years and applications continue to broaden as transposon-based techniques improve. Recent studies of a number of murine and human cancer cell lines have established the efficacy of both Non-Nucleoside and Nucleoside Reverse Transcription Inhibitors (NRTIs) in suppressing L1 retrotransposition, inducing significant cell growth and differentiation phenotypes [65], [66]. Collectively, these studies suggest that L1 retrotransposases may be highly effective as molecular targets in cancer therapy.

The identification of ∼600 bp of a *RAS* related leukemia viral oncogene (LOC642550) proximal to the L1 retrotransposon within the *NAV3 -SYT1-PAWR* gene cluster is also of interest given the known and shared role of rearrangement mediated FUS gene fusions between myxoid liposarcoma and leukemia [67]–[71]. Although the rearrangement hotspot and gene fusions identified in our studies are different, the presence of a pseudogene with homology to a *RAS* related leukemia viral oncogene within the identified hotspot is suggestive that perhaps gene fusions involving *NAV3-SYT1-PAWR* may also play a yet unidentified role in other cancers in addition to well-differentiated liposarcoma.

Structural variants resulting in potential fusion genes are often associated with amplified genes. This work identified amplification in two genes of particular interest. The first is *SYT1,* a calcium sensor involved in conducting neurotransmitter signals, which contained structural rearrangements with three different genes and is possibly part of a ring chromosome, commonly present in WDLS. Interestingly, a 40-kDa form of SYT1 participates in the non-classical export of FGF1, a protein that regulates many cellular processes including angiogenesis, morphogenesis and tumor growth suggesting a potentially key role of SYT1 in cancer [72]. While the precise role of *SYT1* in cancer is unclear, the presence of multiple chromosomal rearrangements within this gene suggests that this gene is prone to breakage. Consequently, mechanistic studies are needed to further elucidate the potential role of *SYT1* and its chromosomal rearrangements in cancer.

Another amplified gene of interest is *DDR2*. Mutations and altered expression in *DDR2* have been reported in multiple tumor types [41]–[47]. *DDR2* is involved in several key roles including proliferation, migration, adhesion and remodeling of the extracellular matrix [48], [49], and is expressed in stromal cells while a relative, *DDR1*, is expressed in epithelial cells [73]. *In vitro* studies of hepatic stellate cells and skin fibroblasts have demonstrated a drop in cell proliferation and migration in the absence of *DDR2* [74]–[76]. Nude mice that received intrasplenic injections of human melanoma cell line A375 with stably silenced *DDR2* had fewer hepatic metastasis than mice receiving mock transfected cells [74]. In contrast *DDR2* ^−/−^ mice that received intrasplenic injection of murine colon adenocarcinoma cell line MCA38 had more hepatic metastasis than equivalently treated *DDR2* ^+/+^ mice [77]. The observed differences in metastatic burden in these mice may be attributable to differing genetic backgrounds, in particular expression of *DDR1* that was not reported in either study. *DDR1* levels have been demonstrated to be inversely proportional to *DDR2* levels, both *in vitro* and *in vivo* [47], [78]. While these studies suggest *DDR2* may play an important role in liposarcoma, its precise role remains to be elucidated through characterization of additional patients with WDLS and by correlating clinical phenotypes with *DDR2* status.

Targeted therapy has enabled tumors with known genetic mutations to be effectively treated by utilizing compounds that exploit a specifically dysregulated molecular pathway. *DDR2*, which is potentially interesting mechanistically, is also a therapeutic target of interest. Imatinib, nilotinib and dasatinib have been demonstrated to inhibit DDR2 kinase activity [50]. Dasatinib effectively inhibited proliferation in cell lines containing *DDR2* mutations [42]. Cells containing *PDGFRA* amplification or *KRAS* mutation were less sensitive to dasatinib and imatinib than cells without these genetic aberrations. In addition, a squamous cell lung cancer patient with a *DDR2* mutation and no *EGFR* mutation demonstrated partial response to dasatinib and erolotinib [42] while a second patient with co-occurring CML and squamous cell lung cancer, which possessed a *DDR2* mutation, showed a complete metabolic response in the lung tumor after treatment with dasatinib [79]. While this data is preliminary, it does suggest that dasatinib may have been a consideration for this WDLS patient with amplified *DDR2*, and thus likely amplified DDR2 kinase activity.

A large amplification of *MDM2* was identified in this patient and is possibly the result of an unidentified gene fusion or the presence of *MDM2* on double minute chromosomes. Interestingly, this patient also had amplification of *CPM*, which when co-occurring with amplified *MDM2* is a unique marker of WDLS [17]. Several MDM2 inhibitors are currently in clinical trials including RO5045337 and RO5503781 (clinicaltrials.gov) of which the first is in a trial targeting liposarcoma. Taken together, the combination of aCGH and WGS allowed the detection of potentially druggable targets in this patient.

While these findings are limited by a sample size of one, this work reveals the value of utilizing multiple technologies to thoroughly interrogate a tumor genome; thus enabling the identification of druggable targets for which therapies are currently available, but are not part of the standard of care for liposarcoma. The cost and time required for next generation sequencing has dropped significantly in recent years along with improvements in variant detection methods, putting work such as this reported here on the brink of clinical application.

In summary, this work is the first to report the entire genome of a WDLS patient utilizing flow cytometry to isolate aneuploid cells prior to aCGH and WGS. We report the identification of a retrotransposon in a hotspot of genomic rearrangement as well as multiple novel structural rearrangements in the genome that likely contribute to the extensive gene amplification observed. In addition, we identified two potential therapeutic targets, MDM2 and DDR2. Further study of these findings in a larger cohort of liposarcoma patients is warranted to estimate the true prevalence of therapeutic targets such as DDR2 and to advance the understanding of the genetic basis of liposarcoma.

## Supporting Information

Figure S1 **Flow cytometry histogram.**
(TIF)

Table S1 **Fusion gene DNA validation primers.**
(DOC)

Table S2 **Bacterial Artificial Chromosomes (BACs) utilized in FISH assays.**
(DOC)

Table S3 **Summary of identified single nucleotide variants.**
(XLS)

Table S4 **Putative fusions identified from whole genome sequencing.**
(XLSX)

Table S5 **Putative fusions identified from RNA sequencing fusion analysis.**
(XLSX)
