# Supplementary material for: Whole Genome Analyses of a Well-Differentiated Liposarcoma Reveals Novel SYT1 and DDR2 Rearrangements
Source: PLoS One. 2014 Feb 5;9(2):e87113. doi: 10.1371/journal.pone.0087113 (PMC3914808; doi:10.1371/journal.pone.0087113)
Supplement: Table S1 — Fusion gene DNA validation primers. (DOC) [file pone.0087113.s002.doc]

| Table S1. Fusion gene DNA validation primers | | | | | |
| --- | --- | --- | --- | --- | --- |
| **Breakpoint 1** | **Gene 1** | **Breakpoint 2** | **Gene 2** | **Forward** | **Reverse** |
| **Intrachromosomal rearrangements** | | | |  |  |
| 1:160738159 | UHMK1 | 1:160878664 | DDR2 | GTCTGTTGTCCATACTCCTC | GGAGAAACCAAATCTGGGGTA |
| 12:70553166 | TBC1D15 | 12:81370954 | C12orf26 | GAGCCATGCACCGTATATTGAATAT | AAAGTGAACGAAGCCAGTCAC |
| 12:77820664 | SYT1 | 12:94015001 | FGD6 | GTCAGTAATAACAGCCCTCAC | CCCTTGGCAATTCATCCCAGTC |
| 12:78116536 | SYT1 | 12:81379417 | C12orf26 | GAATACTAAGGATGGCCTTATG | CTTCTTCATGCCTGTCTCTG |
| 12:78600788 | PAWR | 12:94875477 | AMDHD1 | CAATGTCGTTCAGCCATGGT | GACCACACTAAGACTTTGGAGG |
| 12:81369919 | C12orf26 | 12:94014762 | FGD6 | AGCCAACTCTGCTAAATGCTTAGC | CACAAAAGGAATATGGGGTGTG |
| 12:81371116 | C12orf26 | 12:94875230 | AMDHD1 | GATCCCTTAGCCTCATCAGTC | CCTCTGTGTTACAGGTAAACAGC |
|  |  |  |  |  |  |
| **Translocations** |  |  |  |  |  |
| 6:157853571 | ZDHHC14 | 12:78591928 | PAWR | F1: CACATGGCAAGTAAGTGTGG  F2: CCTCATATAAGCACTTCACAAGG | R1: CCTGAGGTTAGGAAACATATGC  R2: CCAAGGCTGATGTTCAGATG |
| 11:21368102 | NELL1 | 12:77114108 | NAV3 | ACACCAAGAGGAACCACTCT | TCTGTCTGTAACACCACTGCTGGA |
| 11:21368605 | NELL1 | 12:78114565 | SYT1 | F1: AGCAGGTCTGGAAAACTCACA  F2: TCAGGTCGTTTGCCCTTCTT  F3: ATTGCCTTGATAATTAGTGGTA | R1: GCAGAGAATCTGAGGTTGAC  R2: CAGACACATAGACGATGAAAC |
| 11:71472153 | LRTOMT | 12:81346827 | C12orf26 | F1: GGACTTGGATAGGCTGCAAG  F2: TGCAAGGGCTCTATGAGTCC  F3: GAGTCCCTAGGACTTGGATA | R1: GAGGACCAAGATTCAACCTG  R2: ATACTACAAAGGAGAGGGCC  R3: GGTGCCAAAAGGAGGACCAA |
| Fusions requiring multiplex PCR reactions to capture the region include the forward (F1-F3) primers and reverse (R1-R3) primers in the multiplex reaction | | | | | |
