# Supplementary material for: Whole Genome Analyses of a Well-Differentiated Liposarcoma Reveals Novel SYT1 and DDR2 Rearrangements
Source: PLoS One. 2014 Feb 5;9(2):e87113. doi: 10.1371/journal.pone.0087113 (PMC3914808; doi:10.1371/journal.pone.0087113)
Supplement: Table S2 — Bacterial Artificial Chromosomes (BACs) utilized in FISH assays. (DOC) [file pone.0087113.s003.doc]

| Table S2. Bacterial Artificial Chromosomes (BACs) utilized in FISH assays | | | |
| --- | --- | --- | --- |
| **Gene** | **BAC** | **Band** | **Label** |
| DDR2 | RP11-646D10 | 1q23.3 | SpectrumOrange |
| CKS1B | RP11-307C12 | 1q21.3 | SpectrumGreen |
| MDM2 | RP11-775J10 | 12q15 | SpectrumOrange |
| SYT1 | RP11-194P14 | 12q21.2 | SpectrumOrange |
| CEP12 | Vysis #32-112012 | 12p11.1-q11 | SpectrumGreen |
